# Maternal BMI changes from the prepregnancy to postpartum period are associated with postpartum cardiometabolic risk factors: a longitudinal study

**DOI:** 10.1007/s00404-023-07154-x

**Published:** 2023-08-01

**Authors:** Huafan Zhang, Linlin Wu, Xiaoxia Wu, Yixuan Chen, Fu-Ying Tian, Aiqi Yin, Fengqiao Hu, Jianing Tong, Xuna Huang, Yanmei Wan, Jianmin Niu

**Affiliations:** 1 Department of Obstetrics, Shenzhen Maternity and Child Healthcare Hospital, The First School of Clinical Medicine, Southern Medical University, Shenzhen, 518028 China; 2 Department of Obstetrics and Gynecology, The Eighth Affiliated Hospital, Sun Yat-sen University, Shenzhen, 518033 China

**Keywords:** Total BMI change, Cardiometabolic risk factors, Longitudinal study, Obesity

## Abstract

**Purpose:**

This study aimed at investigating the associations between the total body mass index (BMI) change at 3 or 4 years postpartum compared to the prepregnancy and cardiometabolic risk factors.

**Methods:**

This longitudinal study included 1305 participants. Based on the total postpartum BMI changes, they were divided into < 0 units, 0–1.7 units, and > 1.7 units groups using the interquartile range. Multiple linear regression models were used to analyze the associations.

**Results:**

Compared to the reference group, there was a progressive increase in the *β*coefficient (*β*coef) of homeostasis model assessment of insulin resistance (HOMA-IR) of cardiometabolic risk in the following groups: the ‘0–1.7 units’ group with the ‘overweight traj’ [*β*coef 0.33; 95% confidence intervals (CI) 0.22, 0.44)] or the ‘obesity traj’ [0.66; (0.45, 0.88)] and the ‘> 1.7 units’ group with the ‘normal traj’ [0.33; (0.22, 0.44)], the ‘overweight traj’ [0.54; (0.41, 0.67)] or the ‘obesity traj’ [0.97; (0.79, 1.15)]. The same increasing trend of *β*coef was also found in DBP, FPG, LDL, WHR, BF%. However, the ‘< 0 units’ group with the ‘low traj’ [0.13; (0.06, 0.21)] and the ‘0–1.7 units’ group with the ‘low traj’ [0.08; (0.03, 0.13)] had higher high-density lipoprotein cholesterol (HDL-C) level than the reference group.

**Conclusion:**

Women with a postpartum BMI gain > 1.7 units are positively associated with cardiometabolic risk factors, especially for those in the ‘obesity traj’ or ‘traj D’. Conversely, women with a postpartum BMI loss > 0 units have negative association with cardiometabolic risk factors, especially for those in the ‘low traj’ or ‘traj B’.

## What does this study add to the clinical work

**Table:** 

To further investigate the correlation between BMI changes from the prepregnancy to postpartum period and cardiometabolic risk factors, taking into account the effects of prepregnancy BMI and gestational weight gain (GWG). In clinical terms, it gives Chinese women a reference for maternal weight recovery after delivery.	

## Introduction

In 2014, China became the country with the highest number of women who are slightly obese worldwide [1]. And even for severe obesity, the number of Chinese women got to 2nd rank [1]. Obesity, which negatively impacts women’s health, has much wider and long-lasting consequences and is generally taken seriously by either health professionals or women themselves [2]. For reproductive-aged women, one of the risk factors for obesity is pregnancy [3–5], possibly through the mechanism of postpartum weight retention or weight gain [6–9]. Cardiometabolic risk is the leading contributor to the disease burden for Chinese women [10]. Obesity is a modifiable risk factor of cardiovascular disease [11], and more than two-thirds of deaths related to high body mass index (BMI) are due to cardiovascular disease [12]. Moreover, postpartum weight gain may cause abdominal adiposity in some women [13], which increases the risk of cardiovascular and metabolic diseases [13, 14]. Therefore, research on the relationship between maternal BMI changes from the prepregnancy to postpartum period and cardiometabolic risk is of great significance to Chinese women’s health.

Similarly, considering both prepregnancy BMI and excessive gestational weight gain (GWG) as risk factors for postpartum weight retention or weight gain [6, 8, 9, 15], we also evaluated their effects on the total postpartum BMI changes. In contrast to the classification criteria used in previous studies: international prepregnancy BMI classification, Institute of Medicine's GWG-recommended classification [16], or self-classified GWG classification [15], we dynamically account for changes in weight from prepregnancy through pregnancy in the form of trajectories. Because even if women have the same prepregnancy BMI or GWG, there might be different subgroups, and gestational weight is always in a dynamic change due to changeable pregnancy situation, which may potentially affect the postpartum weight, and then affects assessing cardiometabolic risk. To date, there are limited studies that can simultaneously account for prepregnancy BMI, GWG, postpartum weight changes, and postpartum cardiovascular risk [16, 17]. To the best of our knowledge, no studies have explored postpartum BMI changes considering the effects of the prepregnancy-to-pregnancy weight trajectory and postpartum cardiometabolic risk factors. Thus, the current study aimed to answer this question.

## Methods

### Study population

**Fig. 1:**
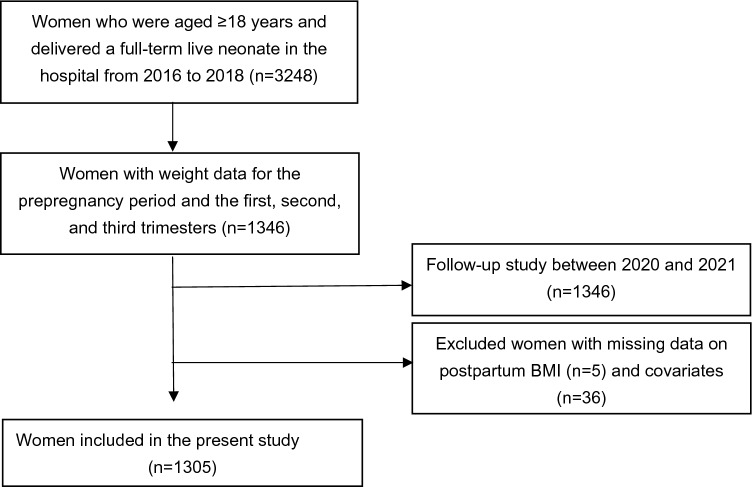
Flowchart of follow-up of cardiometabolic health

We conducted a longitudinal study included 1346 pregnant women aged ≥ 18 years who delivered a full-term live neonate at Shenzhen Maternity & Child Healthcare Hospital, a government hospital in the fourth largest city of China, between 2016 and 2018 and had weight data for the prepregnancy period and the first, second, and third trimesters. A follow-up study was conducted between 2020 and 2021, that is, at 3 or 4 years postpartum, to collect data on basic information and outcome variables. We excluded women with missing data on postpartum BMI (*n* = 5, 0.37%) and covariates (*n* = 36, 2.67%). Ultimately, 1305 women were included in the present study (Fig. 1). The Shenzhen Maternity & Child Healthcare Hospital Research Ethics Board approved the study protocol, and all participants signed informed consent forms.

### Exposure variables

#### Total BMI change from the prepregnancy to postpartum period

We calculated the participants’ difference of BMI [weight (kg)/height (m^2^)] units between the postpartum and prepregnancy and divided them into three groups using the interquartile range (IQR)as follows: the postpartum BMI loss group: < 0 units; the reference group: the first quantile to the third quartile, or 0–1.7 units; and the postpartum BMI gain group: > 1.7 units. Prepregnancy BMI was obtained from the hospital medical system. Postpartum BMI was measured by trained research assistants or nurses at the 3- or 4-year postpartum interview.

#### Prepregnancy-to-pregnancy BMI trajectories

We chose four points to model BMI trajectories, including the prepregnancy BMI, first-trimester BMI, second-trimester BMI, and third-trimester BMI. Considering that pregnant women visited in different weeks during every trimester, we believed that as long as there was one BMI data for the first (week 10 to week 13), second (week 24 to week 27) and third (week 36 to week 39) trimesters, it could be regarded as the BMI of the period.

### Outcome variables

#### Cardiometabolic risk factors at 3 or 4 years postpartum

At the 3-year or 4-year postpartum visit, the waist-to-hip ratio (WHR) and body fat percentage (BF%) were measured using a TSINGHUA TONGFANG BCA-1B. Systolic blood pressure (SBP) and diastolic blood pressure (DBP) were measured 2 times, 2 min apart, with an OMRON HBP-9020; then, the average SBP and DBP were calculated and were included in the final models. Blood was collected to measure biochemical data. Fasting plasma glucose (FPG) was assayed by a hexokinase/glucose-6-phosphate dehydrogenase method, and fasting insulin was measured with a microparticle enzyme immunoassay. Then, the homeostasis model assessment of insulin resistance (HOMA-IR) [fasting insulin (µU/mL) × fasting plasma glucose (mmol/L)/22.5] was calculated. The triglyceride (TG) and total cholesterol (TC) levels were assayed by enzymatic methods. Low-density lipoprotein cholesterol (LDL-C) level was measured with a homogenous direct method, and high-density lipoprotein cholesterol (HDL-C) level was measured using a direct enzymatic colorimetric assay. Since carotid intima-media thickness (CIMT) was shown to predict cardiovascular risk in multiple large studies [18, 19] and associated with cardiovascular disease in Asians [20], we also included the maximum CIMT (CIMT max) measured using a Phillips-SD 800 7.5 MHz transducer as a cardiometabolic risk factor. All of the above data were measured using the standardized protocol by trained research assistants or nurses.

### Covariates

The women’s sociodemographic information, including age, education level, and parity, was obtained. Information on diabetes, gestational diabetes mellitus and pregestational diabetes mellitus, hypertensive disorders of pregnancy, and infant’s gender was obtained from the hospital medical system. Information on smoking habits and alcohol intake was also provided but did not alter the *β*coefficients (*β*coef) or 95% confidence intervals (CI) and were not included in the final models.

### Statistical analysis

We used 4 points as mentioned above to identify prepregnancy-to-pregnancy BMI trajectories using latent class growth modelling (LCGM) [21]. LCGM, an example of finite mixture models, is a method of modeling developmental trajectories that classify individuals within a population that fall into specific clusters. The estimation of the model parameters is achieved by maximum likelihood. The shape and number of the final trajectory groups are based on five aspects, including a priori knowledge, the Bayesian information criterion (BIC), choice of polynomial terms, the average posterior probability (≥ 0.70 for each group), and parsimony in the number of trajectory groups. See “Traj” package in STATA for more details. Ultimately, we obtained 4 trajectories and named them from bottom to top as follows: the ‘low traj’, ‘normal traj’, ‘overweight traj’, ‘obesity traj’**.**

To estimate the associations between the total BMI change and cardiometabolic risk factors at 3 or 4 years postpartum, we used multiple linear regression (MLR) models to estimate the *β*coef and 95% CI. To explore how gestational BMI trajectory groups modify the association between the total BMI change and cardiometabolic risk factors, we evaluated how permutations of the gestational BMI trajectory groups and the total BMI change in 12 groups were associated with cardiometabolic risk factors while simultaneously considering Bonferroni’s correction (corrected *p* < 0.005). Women in the ‘0–1.7 units’ group with the ‘normal traj’ served as the reference group. The models were adjusted for sociodemographic information, diabetes, hypertensive disorders of pregnancy, and infant’s gender.

Considering that women with different prepregnancy BMI is recommended for corresponding GWG, there may be underlying trend trajectories. To eliminate this potential effect, we chose the group of women with a normal prepregnancy BMI and then set up a permutation of the trajectories and the total BMI change for further MLR with cardiometabolic risk factors.

All analyses were conducted in Stata MP (version 16).

## Results

Table 1 summarizes the sociodemographic, obstetrical and follow-up information of the study participants. These characteristics of the women were comparable among each total BMI change group. The women were of a mean age of 31.9 years at enrollment, and most of them had a college education (78.3%), had a normal prepregnancy BMI (70.1%), had given birth only once (56.4%), and had no hypertensive disorders of pregnancy (98.8%) or diabetes during pregnancy (80.7%). The means of the postpartum physical examination results were all normal. Women in the ‘> 1.7 units’ group (mean age 31.3 years) were younger and accounted for 24.5% of the sample, and women in the ‘< 0 units’ group (mean age 32.1 years) were older and accounted for 24.1% of the sample compared with the ‘0–1.7 units’ group (mean age 32.0 years).

**Table 1:** Characteristics of 1305 women by BMI changes from the prepregnancy to postpartum period in a longitudinal study

	ALL	Total BMI change from prepregnancy to postpartum period				
		< 0	0–1.7	> 1.7	*p*	Missing
Characteristics	1305	*n* = 315(24.1%)	*n* = 671(51.4%)	*n* = 320(24.5%)		
Age (years)	31.86 ± 4.18	32.05 ± 3.81	32.04 ± 4.35	31.29 ± 4.13	0.02	0
College graduate					0.136	0
Yes	1022 (78.3)	259 (82.2)	519 (77.3)	244 (76.2)		
No	284 (21.7)	56 (17.8)	152 (22.7)	76 (23.8)		
Parity					0.019	0
0	532 (40.7)	116 (36.8)	260 (38.7)	156 (48.8)		
1	737 (56.4)	190 (60.3)	390 (58.1)	157 (49.1)		
2+	37 (2.8)	9 (2.9)	21 (3.1)	7 (2.2)		
Infant's gender					0.143	0
Female newborns	604 (46.2)	157 (49.8)	293 (43.7)	154 (48.1)		
Male newborns	702 (53.8)	158 (50.2)	378 (56.3)	166 (51.9)		
Prepregnancy BMI (kg/m^2^)^a^					< 0.001	0
< 18.5	151 (11.6)	20 (6.3)	92 (13.7)	39 (12.2)		
18.5–23.9	916 (70.1)	210 (66.7)	479 (71.4)	227 (70.9)		
24–27.9	188 (14.4)	68 (21.6)	84 (12.5)	36 (11.2)		
≥ 28	51 (3.9)	17 (5.4)	16 (2.4)	18 (5.6)		
Postpartum BMI (kg/m^2^)^a^					< 0.001	0
< 18.5	96 (7.4)	43 (13.7)	51 (7.6)	2 (0.6)		
18.5–23.9	855 (65.5)	219 (69.5)	475 (70.8)	161 (50.3)		
24–27.9	278 (21.3)	43 (13.7)	124 (18.5)	111 (34.7)		
≥ 28	77 (5.9)	10 (3.2)	21 (3.1)	46 (14.4)		
Hypertensive disorders of pregnancy					0.404	0
Yes	16 (1.2)	6 (1.9)	6 (0.9)	4 (1.2)		
No	1290 (98.8)	309 (98.1)	665 (99.1)	316 (98.8)		
Diabetes during pregnancy					0.002	0
Yes	252 (19.3)	74 (23.5)	137 (20.4)	41 (12.8)		
No	1054 (80.7)	241 (76.5)	534 (79.6)	279 (87.2)		
SBP (mmHg)	107.91 ± 11.26	107.64 ± 12.50	107.15 ± 10.75	109.80 ± 10.85	0.002	2
DBP (mmHg)	69.67 ± 8.40	69.68 ± 8.97	68.96 ± 8.04	71.15 ± 8.38	0.001	2
FPG (mmol/L)	4.88 ± 0.47	4.82 ± 0.45	4.85 ± 0.41	5.00 ± 0.59	< 0.001	114
HOMA-IR	1.20 ± 1.04	0.95 ± 0.77	1.08 ± 0.82	1.68 ± 1.45	< 0.001	151
TC (mmol/L)	4.61 ± 0.89	4.60 ± 0.83	4.56 ± 0.92	4.74 ± 0.87	0.015	102
TG (mmol/L)	0.87 ± 1.00	0.80 ± 1.42	0.81 ± 0.74	1.04 ± 0.94	0.002	98
HDLC (mmol/L)	1.43 ± 0.31	1.48 ± 0.32	1.44 ± 0.31	1.34 ± 0.29	< 0.001	106
LDLC (mmol/L)	2.67 ± 0.73	2.62 ± 0.70	2.61 ± 0.72	2.84 ± 0.75	< 0.001	106
BF%	26.65 ± 6.60	25.22 ± 6.54	29.52 ± 6.07	25.95 ± 6.48	< 0.001	1
WHR	0.80 ± 0.06	0.80 ± 0.05	0.80 ± 0.05	0.82 ± 0.06	< 0.001	1
CIMT max	0.61 ± 0.08	0.61 ± 0.07	0.61 ± 0.09	0.61 ± 0.08	0.624	14
Trajectory groups					< 0.001	0
‘low traj’	374 (28.6)	78 (24.8)	225 (33.5)	71 (22.2)		
‘normal traj’	515 (39.4)	113 (35.9)	269 (40.1)	133 (41.6)		
‘overweight traj’	330 (25.3)	97 (30.8)	151 (22.5)	82 (25.6)		
‘obesity traj’	87 (6.7)	27 (8.6)	26 (3.9)	34 (10.6)		

Data are showed as median (IQR) or mean (SD)

*IQR* interquartile range, *SD* standard difference, *BMI* body mass index, *SBP* systolic blood pressure, *DBP* diastolic blood pressure, *FPG* fasting plasma glucose, *HOMA-IR* homeostasis model assessment of insulin resistance, *TC* total cholesterol, *TG* triglyceride, *HDL-C* high-density lipoprotein cholesterol, *LDL-C* low-density lipoprotein cholesterol, *BF%* body fat percentage, *WHR* waist-to-hip ratio, *CIMT max* the maximum of carotid intima-media thickness

^a^WHO concludes that the BMI of the Chinese population should be reduced within 3 units from the current WHO cut-off point of 25 kg/m^2^ for observed risk, so we used the cut-off points developed by the Working Group of Obesity, China [22–24]

### Prepregnancy-to-pregnancy BMI trajectories

**Fig. 2:**
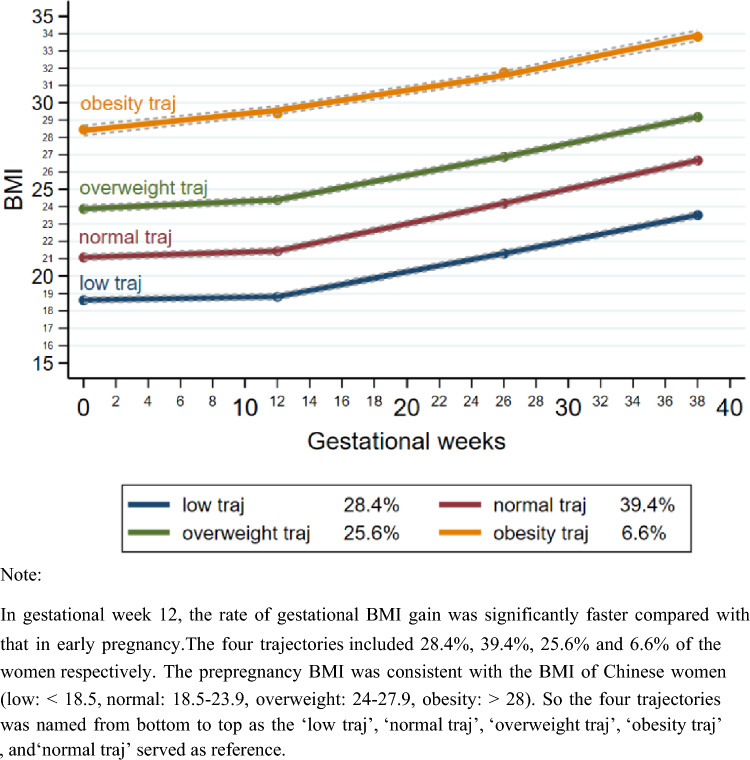
Prepregnancy-to-pregnancy BMI trajectories among 1305 women

The four trajectories are shown in Fig. 2. In mid-pregnancy, the rate of gestational BMI gain was significantly faster compared with that in early pregnancy, and the rates of gestational BMI gain in the first three trajectories slowed in late pregnancy according to final polynomial terms. The first three trajectories included 28.4%, 39.4% and 25.6% of the women, respectively. Only the ‘obesity traj’ maintained a constant weight gain rate from mid-pregnancy onward and included 6.6% of the women. Furthermore, the starting points of the four trajectories, the prepregnancy BMI, were 18.57, 21.08, 23.93, 28.47, and it seemed to be consistent with the BMI of Chinese women (low: < 18.5, normal: 18.5–23.9, overweight: 24–27.9, obesity: > 28) [24]. So we named the four trajectories from bottom to top as the ‘low traj’, ‘normal traj’, ‘overweight traj’, ‘obesity traj’, and ‘normal traj’ served as reference.

### Associations between the total BMI change and cardiometabolic risk factors

Table 2 shows the associations between the total BMI change from the prepregnancy to postpartum period and cardiometabolic risk factors stratified by BMI trajectory groups. Compared to the reference group, there was a progressive increase in the *β*coef of HOMA-IR of cardiometabolic risk in the following groups: the ‘0–1.7 units’ group with the ‘overweight traj’ [*β*coef 0.33; 95% CI 0.22, 0.44)] or the ‘obesity traj’ [0.66; (0.45, 0.88)] and the ‘> 1.7 units’ group with the ‘normal traj’ [0.33; (0.22, 0.44)], the ‘overweight traj’ [0.54; (0.41, 0.67)] or the ‘obesity traj’ [0.97; (0.79, 1.15)] in the postpartum period. The same increasing trend of *β*coef was also found in DBP, FPG, LDL, WHR, BF%. However, the ‘< 0 units’ group with the ‘low traj’ [0.13; (0.06, 0.21)] and the ‘0–1.7 units’ group with the ‘low traj’ [0.08; (0.03, 0.13)] had higher HDL-C level than the reference group. These two groups also had lower WHR and BF% values than the reference group. For adiposity factors, the WHR and BF% were statistically significant in almost all groups. The ‘0–1.7 units’ group with the ‘obesity traj’ had a higher CIMT max [0.06 (0.03, 0.10)] than the reference group.

**Table 2:** *β*coef and 95% CI for associations between the total BMI change and cardiometabolic risk factors stratified by BMI trajectories (*n* = 1305)

Total BMI change 0–1.7				Total BMI change > 1.7			
‘low traj’	‘normal traj’	‘overweight traj’	‘obesity traj’	‘low traj’	‘normal traj’	‘overweight traj’	‘obesity traj’
*β* (95% CI)^a^ *p*				*β* (95% CI)^a^ *p*			
− 0.01 (− 0.04, 0.02) 0.388	Reference	0.00 (− 0.02, 0.04) 0.659	0.03 (− 0.03, 0.09) 0.332	− 0.00 (− 0.05, 0.03) 0.728	0.01 (− 0.02, 0.05) 0.452	0.03 (− 0.01, 0.06) 0.192	0.09 (0.04, 0.15) 0.001
0.37 (− 1.79, 2.53) 0.735	Reference	3.91 (1.48, 6.34) 0.002	4.91 (0.03, 9.78) 0.049	1.36 (− 1.83, 4.55) 0.404	2.25 (− 0.28, 4.77) 0.081	2.94 (− 0.06, 5.94) 0.055	9.81 (5.50, 14.12) 0.000
0.03 (− 0.05, 0.11) 0.507	Reference	0.09 (0.00, 0.19) 0.049	0.04 (− 0.15, 0.22) 0.709	0.09 (− 0.03, 0.21) 0.133	0.14 (0.05, 0.24) 0.003	0.26 (0.14, 0.37) 0.000	0.61 (0.45, 0.76) 0.000
− 0.13 (− 0.23, − 0.04) 0.007	Reference	0.33 (0.22, 0.44) 0.000	0.66 (0.45, 0.88) 0.000	0.07 (− 0.07, 0.21) 0.308	0.33 (0.22, 0.44) 0.000	0.54 (0.41, 0.67) 0.000	0.97 (0.79, 1.15) 0.000
0.05 (− 0.11, 0.22) 0.543	Reference	0.00 (− 0.18, 0.19) 0.978	0.59 (0.22, 0.96) 0.002	0.23 (− 0.01, 0.47) 0.057	0.21 (0.02, 0.40) 0.030	0.36 (0.13, 0.58) 0.002	0.12 (− 0.20, 0.44) 0.459
− 0.03 (− 0.15, 0.09) 0.621	Reference	0.29 (0.15, 0.43) 0.000	0.57 (0.29, 0.84) 0.000	0.13 (− 0.05, 0.30) 0.159	0.35 (0.21, 0.49) 0.000	0.44 (0.27, 0.60) 0.000	0.61 (0.37, 0.84) 0.000
0.08 (0.03, 0.13) 0.004	Reference	− 0.17 (− 0.23, − 0.11) 0.000	− 0.20 (− 0.32, − 0.08) 0.001	− 0.01 (− 0.09, 0.07) 0.734	− 0.15 (− 0.21, − 0.09) 0.000	− 0.10 (− 0.18, − 0.03) 0.008	− 0.29 (− 0.40, − 0.19) 0.000
− 0.02 (− 0.15, 0.12) 0.782	Reference	0.14 (− 0.01, 0.29) 0.068	0.31 (0.01, 0.61) 0.042	0.25 (0.05, 0.44) 0.734	0.24 (0.08, 0.39) 0.014	0.39 (0.20, 0.57) 0.002	0.28 (0.03, 0.54) 0.000
− 0.02 (− 0.03, − 0.00) 0.000	Reference	0.03 (0.02, 0.04) 0.000	0.07 (0.05, 0.08) 0.000	0.00 (− 0.00, 0.02) 0.393	0.01 (0.00, 0.02) 0.006	0.04 (0.03, 0.05) 0.000	0.10 (0.08, 0.12) 0.000
− 12.61 (− 17.51, − 7.71) 0.000	Reference	9.28 (3.78, 14.78) 0.001	24.93 (13.88, 35.99) 0.000	3.88 (− 3.39, 11.16) 0.295	12.59 (6.88, 18.29) 0.000	13.80 (7.00, 20.59) 0.000	36.91 (27.15, 46.68) 0.000
− 0.00 (− 0.02, 0.01) 0.806	Reference	0.01 (− 0.00, 0.03) 0.121	0.06 (0.03, 0.10) 0.000	0.00 (− 0.02, 0.02) 0.821	0.01 (− 0.00, 0.03) 0.142	0.02 (0.00, 0.04) 0.034	0.01 (− 0.01, 0.04) 0.291

Outcome variables	Total BMI change < 0			
	‘low traj’	‘normal traj’	‘overweight traj’	‘obesity traj’
	*Β* (95% CI)^a^ *p*			
SBP^b^	− 0.02 (− 0.06, 0.02) 0.339	− 0.03 (− 0.07, 0.00) 0.052	0.05 (0.00, 0.08) 0.016	0.03 (− 0.03, 0.10) 0.298
DBP	0.83 (− 2.23, 3.89) 0.595	− 0.14 (− 2.80, 2.53) 0.919	3.72 (0.88, 6.55) 0.010	3.13 (− 1.67, 7.94) 0.201
FPG	− 0.11 (− 0.22, 0.01) 0.076	0.00 (− 0.10, 0.10) 0.993	0.07 (− 0.04, 0.18) 0.203	0.04 (− 0.14, 0.23) 0.667
HOMA-IR^b^	− 0.29 (− 0.42, − 0.15) 0.000	− 0.15 (− 0.2, − 0.03) 0.016	0.11 (− 0.02, 0.24) 0.087	0.24 (0.02, 0.46) 0.033
TC	0.13 (− 0.10, 0.36) 0.271	0.11 (− 0.09, 0.31) 0.280	0.05 (− 0.16, 0.27) 0.633	− 0.10 (− 0.45, 0.26) 0.599
TG^b^	− 0.02 (− 0.19, 0.14) 0.782	− 0.04 (− 0.19, 0.11) 0.636	0.02 (− 0.14, 0.18) 0.780	− 0.07 (− 0.33, 0.19) 0.604
HDL-C	0.13 (0.06, 0.21) 0.001	0.06 (− 0.00, 0.13) 0.095	− 0.07 (− 0.14, − 0.00) 0.067	− 0.12 (− 0.24, − 0.00) 0.046
LDL-C	− 0.01 (− 0.19, 0.18) 0.958	0.05 (− 0.12, 0.21) 0.579	0.09 (− 0.09, 0.26) 0.344	− 0.08 (− 0.37, 0.21) 0.604
WHR	− 0.03 (− 0.04, − 0.02) 0.000	− 0.00 (− 0.02, 0.00) 0.093	0.02 (0.01, 0.04) 0.000	0.03 (0.02, 0.05) 0.000
BF%	− 16.88 (− 23.81, − 9.95) 0.000	− 9.89 (− 15.92, − 3.85) 0.001	− 2.23 (− 8.62, 4.17) 0.495	8.59 (− 2.30, 19.47) 0.122
CIMT max	− 0.01 (− 0.03, 0.01) 0.618	− 0.00 (− 0.02, 0.01) 0.601	0.01 (− 0.01, 0.02) 0.563	0.02 (− 0.01, 0.06) 0.122

Adjusted for sociodemographic information, diabetes, hypertensive disorders of pregnancy, and infant’s gender

*βcoef* *β*coefficients, *SBP* systolic blood pressure, *DBP* diastolic blood pressure, *FPG* fasting plasma glucose, *HOMA-IR* homeostasis model assessment of insulin resistance, *TC* total cholesterol, *TG* triglyceride, *HDL-C* high-density lipoprotein cholesterol, *LDL-C* low-density lipoprotein cholesterol, *BF%* body fat percentage, *WHR* waist-to-hip ratio, *CIMTmax* the maximum of carotid intima-media thickness

^a^Multiple linear regression models to estimate *β*coef and 95% CI considering Bonferroni’s correction (corrected *p* < 0.005)

^b^Log-transformed variables before analysis: SBP, HOMA-IR, TG

### Associations between the total BMI change and cardiometabolic health in women with normal prepregnancy BMI

**Fig. 3:**
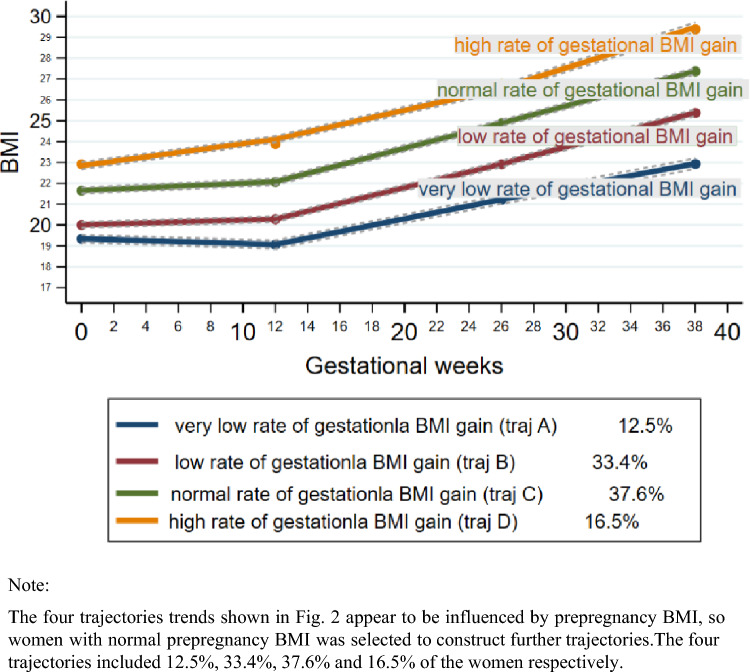
Prepregnancy-to-pregnancy BMI trajectories in women with a normal prepregnancy BMI (*n* = 916)

The four trajectories trends shown in Fig. 2 appear to be influenced by prepregnancy BMI, so we selected women with normal prepregnancy BMI to construct further trajectories and then analyze the relationships between these trajectories and cardiometabolic risk factors. The four trajectories are shown in Fig. 3 and had similar trends as those shown in Fig. 2. The four trajectories included 12.5%, 33.4%, 37.6% and 16.5% of the women respectively. It can be seen from the figure that the rate of gestational BMI gain (traj A: 19.33–22.82; traj B: 19.99–25.36; traj C: 21.68–27.38; traj D: 22.93–29.47) gradually accelerates with the increase of the trajectories. And according to prepregnancy BMI and the rate of gestational BMI gain, we named the four trajectories from bottom to top as follows: the traj A (very low rate of gestational BMI gain traj), traj B (low rate of gestational BMI gain traj), traj C (normal rate of gestational BMI gain traj), traj D (high rate of gestational BMI gain traj). The ‘traj C’ served as the reference group.

The MLR results in Table 3 showed the same direction in further analysis. And women in the ‘0–1.7 units’ group with the ‘traj C’ served as the reference group.

**Table 3:** *β*coef and 95% CI for associations between the total BMI change and cardiometabolic risk factors stratified by BMI trajectories in women with normal prepregnancy BMI (*n* = 916)

Total BMI change 0–1.7				Total BMI change > 1.7			
‘traj A’	‘traj B’	‘traj C’	‘traj D’	‘traj A’	‘traj B’	‘traj C’	‘traj D’
*β* (95% CI)^a^ *p*				*β* (95% CI)^a^ *p*			
− 0.19 (− 3.09, 2.71) 0.897	− 0.61 (− 2.74, 1.52) 0.571	Reference	0.47 (− 2.48, 3.41) 0.756	3.80 (− 1.72, 9.33) 0.177	0.25 (− 2.56, 3.06) 0.861	3.75 (1.11, 6.39) 0.005	2.94 (− 0.10, 5.99) 0.058
0.29 (− 1.95, 2.52) 0.801	− 0.63 (− 2.27, 1.01) 0.454	Reference	0.84 (− 1.43, 3.11) 0.469	2.08 (− 2.18, 6.33) 0.338	0.64 (− 1.53, 2.80) 0.564	3.41 (1.37, 5.44) 0.001	2.51 (0.16, 4.86) 0.036
− 0.02 (− 0.14, 0.09) 0.698	− 0.06 (− 0.15, 0.03) 0.169	Reference	− 0.01 (− 0.12, 0.11) 0.929	0.03 (− 0.19, 0.24) 0.816	0.09 (− 0.02, 0.20) 0.111	0.14 (0.03, 0.25) 0.010	0.16 (0.04, 0.28) 0.011
− 0.10 (− 0.25, 0.05) 0.179	− 0.08 (− 0.19, 0.04) 0.185	Reference	0.18 (0.03, 0.33) 0.020	0.07 (− 0.21, 0.35) 0.608	0.15 (0.01, 0.30) 0.034	0.35 (0.21, 0.48) 0.000	0.43 (0.28, 0.58) 0.000
0.19 (− 0.07, 0.44) 0.150	0.05 (− 0.14, 0.24) 0.596	Reference	− 0.18 (− 0.43, 0.08) 0.177	0.31 (− 0.16, 0.77) 0.198	− 0.01 (− 0.25, 0.24) 0.959	0.40 (0.17, 0.64) 0.001	0.29 (0.03, 0.55) 0.029
− 0.08 (− 0.27, 0.11) 0.400	− 0.10 (− 0.24, 0.04) 0.164	Reference	0.04 (− 0.15, 0.23) 0.679	0.23 (− 0.12, 0.58) 0.198	0.10 (− 0.08, 0.28) 0.268	0.38 (0.21, 0.55) 0.000	0.25 (0.05, 0.44) 0.014
0.13 (0.04, 0.22) 0.003	0.06 (− 0.00, 0.13) 0.056	Reference	− 0.10 (− 0.18, − 0.01) 0.038	− 0.05 (− 0.21, 0.11) 0.549	− 0.07 (− 0.16, 0.01) 0.095	− 0.14 (− 0.22, − 0.06) 0.001	− 0.05 (− 0.14, 0.05) 0.321
0.12 (− 0.09, 0.34) 0.269	− 0.01 (− 0.17, 0.15) 0.894	Reference	0.01 (− 0.21, 0.23) 0.946	0.26 (− 0.13, 0.66) 0.194	0.08 (− 0.12, 0.29) 0.433	0.40 (0.20, 0.59) 0.000	0.27 (0.04, 0.49) 0.021
− 0.02 (− 0.03, − 0.01) 0.004	− 0.01 (− 0.02, − 0.00) 0.014	Reference	0.01 (− 0.00, 0.02) 0.152	0.02 (− 0.01, 0.04) 0.190	0.01 (− 0.01, 0.02) 0.334	0.01 (0.00, 0.02) 0.050	0.03 (0.01, 0.04) 0.000
− 2.76 (− 4.32, − 1.20) 0.001	− 2.03 (− 3.17, − 0.88) 0.001	Reference	1.15 (− 0.43, 2.74) 0.153	2.78 (− 0.19, 5.74) 0.067	0.92 (− 0.59, 2.42) 0.232	1.68 (0.26, 3.10) 0.021	3.81 (2.17, 5.44) 0.000
− 0.01 (− 0.03, 0.01) 0.354	− 0.00 (− 0.02, 0.01) 0.604	Reference	− 0.00 (− 0.02, 0.01) 0.658	− 0.01 (− 0.05, 0.02) 0.477	− 0.01 (− 0.02, 0.01) 0.499	0.02 (− 0.00, 0.03) 0.065	0.01 (− 0.01, 0.03) 0.145

Outcome variables	Total BMI change < 0			
	‘traj A’	‘traj B’	‘traj C’	‘traj D’
	*β* (95% CI)^a^ *p*			
SBP^b^	1.82 (− 2.11, 5.74) 0.364	− 3.48 (− 6.37, − 0.59) 0.018	− 1.96 (− 4.58, 0.65) 0.141	2.80 (− 1.12, 6.73) 0.161
DBP	1.32 (− 1.70, 4.35) 0.390	− 0.63 (− 2.86, 1.60) 0.579	− 0.92 (− 2.94, 1.10) 0.371	2.02 (− 1.01, 5.04) 0.191
FPG	− 0.03 (− 0.19, 0.14) 0.756	− 0.16 (− 0.28, − 0.04) 0.009	− 0.02 (− 0.13, 0.09) 0.725	0.02 (− 0.14, 0.18) 0.792
HOMA-IR^b^	− 0.22 (− 0.43, − 0.01) 0.036	− 0.37 (− 0.52, − 0.22) 0.000	− 0.08 (− 0.21, 0.06) 0.273	− 0.12 (− 0.33, 0.08) 0.230
TC	0.23 (− 0.11, 0.56) 0.188	− 0.03 (− 0.29, 0.22) 0.790	0.13 (− 0.10, 0.36) 0.274	0.01 (− 0.33, 0.36) 0.938
TG^b^	0.01 (− 0.24, 0.27) 0.923	− 0.28 (− 0.48, − 0.09) 0.004	− 0.04 (− 0.21, 0.13) 0.651	− 0.28 (− 0.54, − 0.02) 0.037
HDL-C	0.17 (0.05, 0.29) 0.005	0.14 (0.05, 0.23) 0.003	0.04 (− 0.04, 0.12) 0.344	0.00 (− 0.12, 0.13) 0.940
LDL-C	0.09 (− 0.20, 0.38) 0.542	− 0.11 (− 0.32, 0.11) 0.342	0.08 (− 0.12, 0.28) 0.416	− 0.02 (− 0.32, 0.28) 0.892
WHR	− 0.04 (− 0.05, − 0.02) 0.000	− 0.02 (− 0.04, − 0.01) 0.000	− 0.01 (− 0.02, 0.00) 0.147	0.01 (− 0.01, 0.03) 0.449
BF%	− 5.18 (− 7.29, − 3.08) 0.000	− 3.94 (− 5.50, − 2.39) 0.000	− 2.11 (− 3.51, − 0.70) 0.003	− 0.22 (− 2.33, 1.89) 0.839
CIMT max	− 0.01 (− 0.03, 0.02) 0.608	− 0.02 (− 0.04, − 0.00) 0.027	− 0.00 (− 0.02, 0.02) 0.952	− 0.02 (− 0.04, 0.01) 0.220

Adjusted for sociodemographic information, diabetes, hypertensive disorders of pregnancy, and infant’s gender

*βcoef* *β*coefficients, *SBP* systolic blood pressure, *DBP* diastolic blood pressure, *FPG* fasting plasma glucose, *HOMA-IR* homeostasis model assessment of insulin resistance, *TC* total cholesterol, *TG* triglyceride, *HDL-C* high-density lipoprotein cholesterol, *LDL-C* low-density lipoprotein cholesterol, *BF%* body fat percentage, *WHR* waist-to-hip ratio, *CIMT max* the maximum of carotid intima-media thickness

^a^Multiple linear regression models to estimate *β*coef and 95% CI meanwhile considering Bonferroni's correction (corrected *p* < 0.005)

^b^Log-transformed variables before analysis: HOMA-IR, TG

## Discussion

Based on the results of this longitudinal study, we observed a positive association between the ‘0–1.7 units’ group or ‘> 1.7 units’ group with the ‘normal traj’ or the ‘overweight traj’ or the ‘obesity traj’ and cardiometabolic risk factors. The opposite was found in the ‘< 0 units’ group or ‘0–1.7 units’ group with the ‘low traj’. These associations were consistent in our further MLR analysis among women who had a normal prepregnancy BMI.

Pregnancy is a risk factor for obesity, especially abdominal adiposity, and these physiological alterations may increase cardiometabolic risk when they extend beyond pregnancy and the traditional postpartum period [25–32]. This is consistent with our findings that the higher the postpartum weight is, the higher the WHR and BF%. It has been speculated that postpregnancy obesity may in part contribute to fertility-related low HDL-C levels [25, 27, 28]. We also found the same results. Diana et al. also used LCGM to model weight trajectories during the two years postpartum and then analyzed the association between the weight trajectories and cardiometabolic markers, concluding that women in the little weight loss + the slight gain trajectory group were heavier and had higher central obesity, inflammation and LDL-C levels at 3 years postpartum [17]. This corresponded to our postpartum BMI gain ‘> 1.7 units’ group, and we also observed that this group had central obesity and high LDL-C level. In a large cohort study with 16 years of follow-up, participants were divided into 4 groups based on the total BMI change from the prepregnancy to postpartum period: < − 1, − 1 to 1, > 1 to 2, and > 2 [16]. An increased risk of hypertension and CVD with postpartum weight gain was observed [16]. Considering low HDL-C level and HOMA-IR as two of the cardiovascular risk factors, we obtained that postpartum BMI gain > 1.7 units was associated with low HDL-C level and high HOMA-IR, and thus we have a possible explanation that postpartum BMI gain increased potential cardiometabolic risk. Hayfaa et al. categorized participants into different levels according to their postpartum weight retention (weight retention < 3 kg; weight retention 3 to < 7 kg; and weight retention ≥ 7 kg) to explore the association between different weight retention levels at 12 months postpartum and cardiometabolic risk [33]. They concluded that cardiometabolic risk augmented with the increase of postpartum weight [33]. Taking into account ethnicity differences, our study found a positive association between postpartum BMI gain > 1.7 units and cardiometabolic risk factors. In addition, other studies also characterized postpartum weight in different ways at 12 months postpartum to prove the association with cardiometabolic risk factors [34, 35]. A follow-up study over 15 years found women who failed to lose weight over 4 years postpartum to be in a significant risk of obesity in midlife [36]. Our study also showed that postpartum BMI gain > 1.7 units at 3 or 4 years postpartum was strongly associated with high WHR and BF%. Furthermore, Lorenz et al. indicated that every 0.1 mm increase in the CIMT was associated with a 10–15% increase in the risk of future myocardial infarction [37]. In our study, we observed that the ‘0–1.7 units’ group with the ‘obesity traj’ had a higher CIMT than the reference group.

Unlike the previously described associations between postpartum weight gain and cardiometabolic risk factors, Diana et al. observed the opposite result as follows: women in fast weight loss + slight gain group developed less weight, less central adiposity and lower insulin resistance [17]. The corresponding finding that WHR and BF% decreased with decreasing postpartum weight in the ‘low traj’ was also observed in our study. We also observed high HDL-C level, which is a strong protective factor against CVD [38], in these groups. A nested comparative study investigating how postpartum weight retention affects ox-LDL and serum lipids stratified participants into tertiles, including weight loss, unaltered weight and weight gain groups. They reported the same results, with women who lost weight having lower TG and ox-LDL/HDL-C level ratios than women who gained weight [39]. Discussing the relationships among three weight loss interventions and cardiometabolic risk factors, including blood pressure, lipids, glucose and insulin resistance, an investigation revealed that enhanced brief lifestyle counseling, which led to the most weight loss, was associated with the largest improvements in HDL-C level and TG levels [40]. Furthermore, other studies found that postpartum weight loss appeared to be associated with the improvement of cardiometabolic risk factors [41–45].

### Strengths and limitations

The major strength of this study is that it is the first large population-based study on the association between the total BMI change from the prepregnancy to postpartum period and postpartum cardiometabolic risk factors in Chinese. This study is essential and informative, as the number of women with obesity in China is increasing with socioeconomic development; the cardiovascular risks involved are also increasing [2, 10, 46]. Another advantage is that to prevent potential established trends in all women, further analysis was performed in participants with normal prepregnancy BMI. Furthermore, the acquisition of data on our exposure and outcome variables was performed by trained and experienced nurses or assistants, which reduced the risk of bias. Our study on cardimetabolic risk factors also applied noninvasive ultrasonography CIMT, which may be a screening method in the future.

There are also some limitations in our study. The major limitation of this study is that because it was a longitudinal study on cardiometabolic risk factors, causality cannot be inferred in the final association between the total BMI change and cardiovascular and metabolic disease. However, we aimed to study a relatively short period of 3 or 4 years postpartum to guide women manage their postpartum weight, which would also reduce to some extent the possible occurrence of cardiovascular disease afterwards. Further, It would be of great interest to perform a prospective follow-up on the occurrence of endpoint events. Although we adjusted for several potential confounders, we could not exclude residual or other unmeasured confounders. Despite these limitations, our data were sufficient to examine the association between the total BMI change from the prepregnancy to postpartum period and cardiometabolic risk factors.

## Conclusion

In conclusion, postpartum BMI gain > 1.7 units is associated with cardiometabolic risk factors, especially for women in the ‘obesity traj’. However, postpartum weight loss > 0 units compared to the prepregnancy weight will contribute to improving cardiometabolic outcomes. Our findings therefore underscore the importance of postpartum weight loss interventions for improving women's health.

## Data Availability

Due to the participants of this study did not agree for their data to be shared publicly.
